# Impact of differential dietary concentrations of cobalt, manganese and zinc on gastrointestinal microbiome and resistome of lactating dairy cattle

**DOI:** 10.1186/s42523-026-00554-9

**Published:** 2026-03-25

**Authors:** Alexandra Langlois, Mélissa Duplessis, Jennifer Ronholm, Antony T. Vincent, Dominic Poulin-Laprade, Renée M. Petri

**Affiliations:** 1 Agriculture and Agri-Food Canada, Sherbrooke Research and Development Center, Sherbrooke, QC Canada; 2 Faculté des sciences de l’agriculture et l’alimentation, Département des sciences animales, Université Laval, Québec, QC Canada; 3 Department of Food Science and Agricultural Chemistry, Faculty of Agricultural and Environmental Sciences, McGill University, Macdonald Campus, Montreal, QC Canada; 4 Department of Animal Science, Faculty of Agricultural and Environmental Sciences, McGill University, Macdonald Campus, Montreal, QC Canada

**Keywords:** Trace mineral supplementation, Metagenomics, Resistome, Antimicrobial resistance genes, Dairy cow gut microbiome

## Abstract

**Background:**

Dietary trace mineral (TM) concentrations for lactating cows often exceed national recommendations under commercial feeding practices. Excess TM supplementation may exert selection pressure on the gut microbiota, promoting metal resistance and potentially co-selecting for important antimicrobial resistance genes (ARGs). This study used a cross-over design to investigate the impact of overfeeding a commericially representative TM premix on the gut microbiome and resistome of lactating dairy cattle. Cows were fed either recommended or surplus TM levels for 31 days followed by sample collection from rumen papillae, whole rumen content, and feces. Targeted amplicon, shotgun metagenomic sequencing, and droplet digital PCR (ddPCR) were used to assess microbial community compositions and the associated resistome.

**Results:**

While a surplus TM did not significantly affect the overall microbial diversity, specific taxa differed between the matrices and to a lesser extent by diet. *Spirochaetota* were more abundant in papillae of cows fed the recommended TM diet, whereas *Bacillota* were more prevalent in the rumen and feces from cows fed the surplus TM. Phosphorus had the greatest impact on prokaryotic taxa in rumen content. Cows fed surplus levels of TM showed increased abundances of *Ruminococcus* in their rumen, but decreased *Campylobacter*, *Desulfovibrio*, and *Treponema* adhered to their rumen papillae, *Methanosphaera* in their rumen and feces, *as well as Treponema* in their feces. Despite these changes, no significant differences in the presence of key ARGs or metal resistance genes were detected by metagenomics, and ddPCR showed no significant impact of TM supplementation on *bla*_CTX−M_, *pcoA* and *zntA* gene levels.

**Conclusions:**

Overfeeding TM in a commercial premix resulted in modest matrix specific shifts I the microbial composition without detectable enrichment of selected ARGs or metal resistance genes. These findings suggest short-term resilience of the rumen ecosystem to surplus TM supplementation. Additionally this work provides foundational knowledge to further guide mechanistic and long-term investigations.

**Supplementary Information:**

The online version contains supplementary material available at 10.1186/s42523-026-00554-9.

## Background

Trace minerals (TM), which include a number of heavy metals, are critical to the support of key metabolic functions and milk production in dairy cows. As such, it is common practice to supplement the diets of North American commercial dairy herds above recommended levels with the goal of optimizing rumen fermentation, production performance, and overall health [1, 2].

However, current nutritional recommendations for TM concentrations in feed do not take into consideration the impact of over-supplementation on the gastrointestinal ecosystem, more specifically the general health and function of the gut microbiome. This is an area of concern as some TMs, such as copper and zinc, are known to exhibit antimicrobial effects [3] that can contribute to the dissemination of antimicrobial resistance (AMR) by enriching resistance gene determinants (metal resistance genes; MRG) via co-selection mechanisms [4], including in livestock animals [5, 6]. This poses additional risks beyond the animal, as TM excreted in the feces could impact soil and water ecosystems [7].

Gastrointestinal bacterial populations in ruminants are essential for the digestion and overall health of cattle, performing the critical tasks of breaking down indigestible feeds into energy forms usable by the cow. As such, these populations can largely be influenced by diet and nutrient availability [8, 9]. To date, many studies have characterized the presence of predicted genes in the microbiota population related to metabolism [10, 11] or studied the impact of TM on health, immunity and productivity of animals [12–15] but less is known about the impact of surplus TM supplementation on the rumen ecosystem population dynamics.

The objective of this study was to assess the relationship between the short-term feeding levels of a commercial sulfate TM, at or above recommended levels, in lactating cow diets and the associated changes in the gut microbiome and resistome with specific focus on cobalt, manganese and zinc. We hypothesized that due to the role of TM in microbial metabolism, surplus feeding would alter the abundance of differential microbial groups and that within the various gut locations, these population dynamics would show variations in copy numbers of specific ARGs and MRGs.

## Methods

### Experimental design, diet and treatments

Experimental procedures were approved by the Institutional Animal Care Committee of the Sherbrooke Research and Development Centre (internal number #575) under the guidelines of the Canadian Council on Animal Care [16] and production related data has already been published by Marchand et al. [17]. Cows were housed in a tie-stall facility at the experimental dairy farm of Agriculture and Agri-Food Canada (Sherbrooke, QC, CA). Animals had been previously cannulated in the rumen. Selection was based on health status, lactation stage, and previous number of lactations. A total of eight multiparous rumen cannulated Holstein cows at 82 ± 10 days in milk were used in a quadruple 2 × 2 cross-over design (*n* = 4 per treatment) over two periods of 31 days each. Animals were fed an isoenergetic and isonitrogenous lactation diet of about 60% forage and 40% concentrate, with mineral supplementation based on body weight and production (650 kg cows, producing 40 kg of milk) [18] for the control diet (Control). The high mineral diet (High) included an increase in total mineral supplementation based on common nutritional practices [17]. The TM concentrations of the diet were analyzed by inductively coupled plasma optical emission spectroscopy (Perkin-Elmer Corp., Norwalk, CT) using the wet acid digestion procedure with 5 mL of nitric acid, 6 mL of hydrogen peroxide, and 2 mL of hydrochloric acid (Mills and Jones Jr., 1996; United States Environmental Protection Agency, 1996). All steps were performed to avoid TM contamination of samples: all containers were acid washed before sampling, and all samples were ground with titanium blades covered with niobium to avoid contamination with studied TM [17]. The analyzed TM composition of both diets is presented in Table 1.

**Table 1:** Measured mineral composition in the diets (mg/kg)

Mineral	Control		SD*	High	SD
Co	0.24		0.05	0.78	0.10
Mn	35		2	115	15
Zn	89		4	168	28
Fe	203		56	205	55
Cu	19		3	19	4
P	3710		147	3901	132
K	12,986		394	12,913	431
Ca	9460		205	9500	236
Mg	2773		92	2685	134

*SD : standard deviation

### Sample collection

After 31 days of diet adaptation, whole rumen content and feces were collected 0 h, 2 h, 4 h and 6 h after feeding, as well as rumen papillae 6 h after feeding, where 0900 h was time zero and feeding occurred immediately after the 0 h sampling time. Whole rumen contents were collected from five points within the rumen (cranial-ventral, cranial-dorsal, caudal-ventral, caudal-dorsal and medial) [19]. A portion of the rumen content was subsampled and frozen at -80 °C for DNA extraction. The remainder of the rumen content was filtered through cheesecloth [20] for the collection of rumen fluid, which was subsampled, snap frozen and stored at -80 °C for DNA extraction. Rumen papillae biopsies were performed by partial rumen evacuation to allow for the rumen wall (25 cm ventral to fistula) to be exteriorized through the fistula. The rumen wall was washed with sterile saline to remove particulate matter, and then biopsies were taken using sterilized scissors, removing approximately 80% of the papillae without damage to lower layers of rumen wall. An area of approximately 1 cm^3^ was collected [21]. Papillae were snap frozen in liquid nitrogen and then stored at -80 °C for DNA extraction. Feces were collected rectally, homogenized, subsampled and frozen at -80 °C. Some of the samples of rumen content and feces were then lyophilized [22] and sent for DNA extraction and sequencing (McGill University, Montreal, QC, Canada).

### Targeted amplicon sequencing and bioinformatic analysis

Targeted amplicon sequencing was performed on whole rumen content, papillae and feces samples (Table 2). DNA extraction from papillae was performed using the PowerSoil DNA isolation kit (Qiagen, ON, Canada), with the addition of mechanical lysis using a double round of bead-beating (Bead Ruptor Elite, Perkin-Elmer, MT, USA) at 6.45 m/s for 60 s with a five minutes incubation on ice between each homogenization step. After elution, all samples were measured for DNA quantity and quality using a Nanodrop (Thermofisher, ON, Canada), and DNA integrity was assessed visually on an agarose gel. All samples were diluted to 25 ng/ul and shipped on dry ice to the sequencing facility (McGill University, Montreal, QC, Canada). Lyophilized samples of whole rumen content and feces were extracted for DNA according to previously published methods [23]. Briefly, samples were subsampled, and then ground in a coffee grinder prior to extraction with the QIAmp DNA Minikit (Qiagen, ON, Canada). DNA library preparation and sequencing were performed as described by Kozich et al. [24]. DNA was subjected to total 16 S rRNA V4 region amplification (515 F: GTGYCAGCMGCCGCGGTAA and 806R: GGACTACHVGGGTWTCTAAT) and prokaryotic amplicons were sequenced using the paired-end sequencing (251 bp) and Illumina MiSeq technology. Analysis of amplicon sequencing data was performed following a QIIME2 SOP [25] that includes the Deblur method as a step to denoise reads into amplicon sequence variants (ASVs). The Additional file S1 describes the sequencing depth for all samples and the number of sequences remaining after quality-score-based filtering and the denoising step using ASVs. Sequences were stitched and cleaned using QIIME2 SOP [25] and the SILVA 138v2025.7v4 database [26]. Calculations of alpha and beta diversity was performed in R using the Phyloseq package (v1.50.0) [27], and visualization with ggplot2 (v3.5.2) [28].

**Table 2:** Distribution of the samples analyzed for each method

	Post-feeding collection time (h)	*N* total
**Targeted amplicon sequencing**		
Rumen content	0, 2, 4, 6	16
Rumen papillae	6	16
Feces	0, 2, 4, 6	16
**Shotgun metagenomics**		
Feces	6	4
**ddPCR**		
Rumen liquid	6	12
Rumen papillae	6	12
Feces	6	12

### Shotgun metagenomics and bioinformatic analysis

All samples were processed for DNA extraction using the double bead-beating combined with a column purification method as described in Zaheer et al. [29] with minor modifications using chemical, mechanical and enzymatic lysis steps. Prior to DNA extractions, feces sampled 6 h after feeding were thawed and pooled, with approximately 0.35 g from each sample, according to the sampling period and the mineral supplementation, representing four pools of four animals (Table 2). Pooled feces were diluted with 0.9% saline (1:2), then transferred to screw-cap tubes containing 0.3 g of 0.1 mm and 0.1 g of 0.5 mm zirconia silicate beads as well as 1 ml of resuspension buffer (600 mM NaCl, 120 mM Tris-HCl, 60 mM EDTA, 200 mM guanidine isothiocyanate) and 5 µl of β-mercaptoethanol (β-ME). Pre-heated (70 °C) 10% SDS (200 µL) was then added. Tubes were thoroughly vortexed for 5 s at maximum speed followed by 3 min at 6.45 m/s on a Bead Ruptor (Omni International, Kennesaw, GA, United Sates). The homogenate was then incubated at 70 °C for 15 min, centrifuged at 4 °C for 15 min at 16,000 × g and 900 µl of the supernatant was transferred to a new tube, then centrifuged at 4 °C for 5 min at 16,000 × g. The supernatant, approximately 800 µl, was transferred to a new tube. A second round of bead beating was performed with resuspension buffer (800 µL), β-ME (5 µL) and 70 °C pre-heated 10% SDS (200 µL) were added to the remaining pellet, mixed, homogenized and centrifuged to collect a second supernatant. The supernatants (lysates) from both homogenization steps were kept separate until column purification as described below. The lysates were mixed with 200 µL of 10 M ammonium acetate, kept on ice for 5 min, and centrifuged at 4 °C for 10 min at 16,000 × g. The supernatants were mixed with an equal volume of isopropanol, placed on ice for 30 min, and centrifuged at 4 °C for 15 min at 16,000 × g. Nucleic acid pellets were washed with 70% ethanol and dried before resuspension in 200 µl TE [10 mM Tris.HCl pH 7.4; 1 mM EDTA]. To further purify metagenomic DNA, dissolved nucleic acids were mixed with 4 µL of DNase-free RNase (10 mg/mL) and incubated at 37 °C for 15 min. Subsequently, 15 µL of proteinase K (20 mg/ mL) and 200 µL of buffer AL (QIAamp Fast DNA Stool Mini Kit; QIAGEN Inc. Toronto, ON, Canada) were added and incubated at 70 °C for 10 min. Following incubation, samples were processed as per manufacturer’s instructions and dried by centrifugation (QIAamp Fast DNA Stool Mini Kit; QIAGEN Inc. Toronto, ON, Canada). Samples were passed twice on the OneStep-96™ PCR Inhibitor Removal Kit (Zymo Research, Irvine, CA, USA) according to the manufacturer instructions for the exception that the prep Solution was centrifuged at 3,500 x g for 8 min and the final elution was performed with a centrifugation at 3,500 x g for 6 min. Purity and concentration of DNA were quantified using a NanoDrop spectrophotometer (Thermo Fisher Scientific). Library preparations and sequencing were conducted by Genome Quebec at the McGill University (Montréal, QC, Canada), using PCR-free shotgun DNA libraries and ran on an Illumina NovaSeq 6000 S4 generating 150 bp paired-end (PE) sequences as per manufacturer’s instructions.

Raw reads were trimmed and decontaminated against a combination of *Bos taurus* (ARS-UCD1.3) and phiX174 (NC_001422) genomes using the quality control modules of a Nextflow metagenomics pipeline named metagenomic_nf (https://github.com/AAFC-Bioinfo-AAC/metagenomic_nf) [see Additional file S2]. Briefly, raw reads undergo a quality control step with FASTP and were aligned to the host reference genomes to exclude contamination by host DNA. Decontamined reads were then assembled with MEGAHIT [30] with the default parameters. Assemblies were binned with METABAT2 [31]. The completeness and contamination levels of bins were assesed with CheckM2 and only those having a degree of completeness higher or equal to 90% and less than 5% of contamination were considered as Metagenome-assembled genomes (MAGs) [32]. In a last step, MAGs were subjected to dereplication with DREP to eliminate redundant genomes.

For the detection of AMR genes, filtered reads issued from the abovementioned workflow were analyzed with the AMR + + pipeline (v3.0.6) along with the MEGARes database (https://github.com/Microbial-Ecology-Group/AMRplusplus) [33]. Read counts that align to AMR genes were normalized using the cumulative sum scaling (CSS). Analysis and visualisations were performed in R version 4.4.3 using the R packages phyloseq v1.50.0 [27], vegan v2.6.10 [34], tidyverse v2.0.0 [35] and their dependencies. Visualisations were created in R using ggplot2 v3.5.1, Polychrome v1.5.1 [36] and ggpubr v0.6.0 [37] and their dependencies.

### Droplet digital PCR

For resistome analysis with droplet digital PCR (ddPCR), DNA from non-lyophilized rumen liquid, papillae, and feces were extracted individually from 12 samples per matrix, representing six animals fed either diet and sampled six hours after feeding (Table 2). The protocol used for DNA extractions was the same as the one used prior to shotgun metagenomics.

ddPCR analysis was conducted by the RNomics Platform, Université de Sherbrooke, Sherbrooke, QC, Canada. Reactions consisted of 10 µl of 2X QX200 ddPCR EvaGreen Supermix (Bio-Rad), 20 ng of DNA, and primer pair solutions (200 nM final). Primer pairs are listed in Table 3. Each reaction mix (20 µl) was converted to droplets with the QX200 droplet generator (Bio-Rad). Droplet-partitioned samples were then transferred to a 96-well plate, sealed and cycled in a C1000 deep well Thermocycler (Bio-Rad) under the following cycling protocol: 95 °C for 5 min (DNA polymerase activation), followed by 50 cycles of 95 °C for 30 s (denaturation), 59 °C for 1 min (annealing) and 72 °C for 30 s (extension) followed by post-cycling steps of 4 °C for 5 min and 90 °C for 5 min (Signal stabilization) and an infinite 12 °C hold. The cycled plate was then transferred and read using the QX200 reader (Bio-Rad) either the same or the following day post-cycling. The concentration is reported in copies/µl of the final 1x ddPCR reaction (using QuantaSoft software from Bio-Rad) [38].

**Table 3:** Primer pairs used in the ddPCR assays

Gene	Primers (5’-3’)	Amplicon size (bp)	Reference
*16 S rRNA*	AGAGTTTGATCMTGGCTCAG	~ 520	[39]
	GWATTACCGCGGCKGCTG		[40]
bla*CTX-M*	ATGTGCAGYACCAGTAARGTKATGGC	336	[41]
	ATCACKCGGRTCGCCNGGRAT		
*pcoA*	GCTGCAGATGGCCAGTATGTAAA	147	[42]
	CCCTCGAGCGTAACCGGTCC		
*zntA*	AGTACGTAACGATGACGTGCTTG	210	[43]
	GTCACCGCTTTGACTTTATCTTCC		

### Statistics

Statistical analyses were performed on alpha diversity and relative abundances of microbial taxa using a PROC MIXED model of SAS (v9.4) [44]. The model consisted of treatment (Control vs. High), matrix (rumen content, rumen papillae, feces) and the interaction between them as the fixed effects, whereas animal and period were considered as random effects. Due to interactions, each matrix was analyzed separately. Comparisons of matrices have been done with a Tukey adjustment on the means of treatments within the matrices for diversity indices. Taxa abundance was not normally distributed; therefore, means were square transformed. Beta-diversity indices were compared using a PERMANOVA with Bray-Curtis dissimilarities. Statistical significance was declared with a *p* < 0.05, and a tendency at 0.05 < *p* < 0.10.

Correlation analysis between measured mineral concentrations (already published [17]) and microbes in each niche was performed using Spearman’s rank correlation with low correlations being ranked at *r* < 0.50, medium at 0.5 ≤ *r* ≤ 0.7, and higher at *r* > 0.7.

Statistical analysis were also conducted on the ddPCR data using SAS (v9.4) [44] with a crossover design. The model consisted of treatment (Control vs. High), matrix (rumen liquid, papillae, feces) and their interaction. Comparisons were performed using repeated measures with the CSH variance-covariance matrix. Statistical significance was declared with a *p* < 0.05.

Resistome comparison between both groups was performed in R using the Maaslin2 package v1.22.0 [45] with a *p* value set as 0.05 for statistical significance.

## Results

### Microbial diversity

**Fig. 1:**
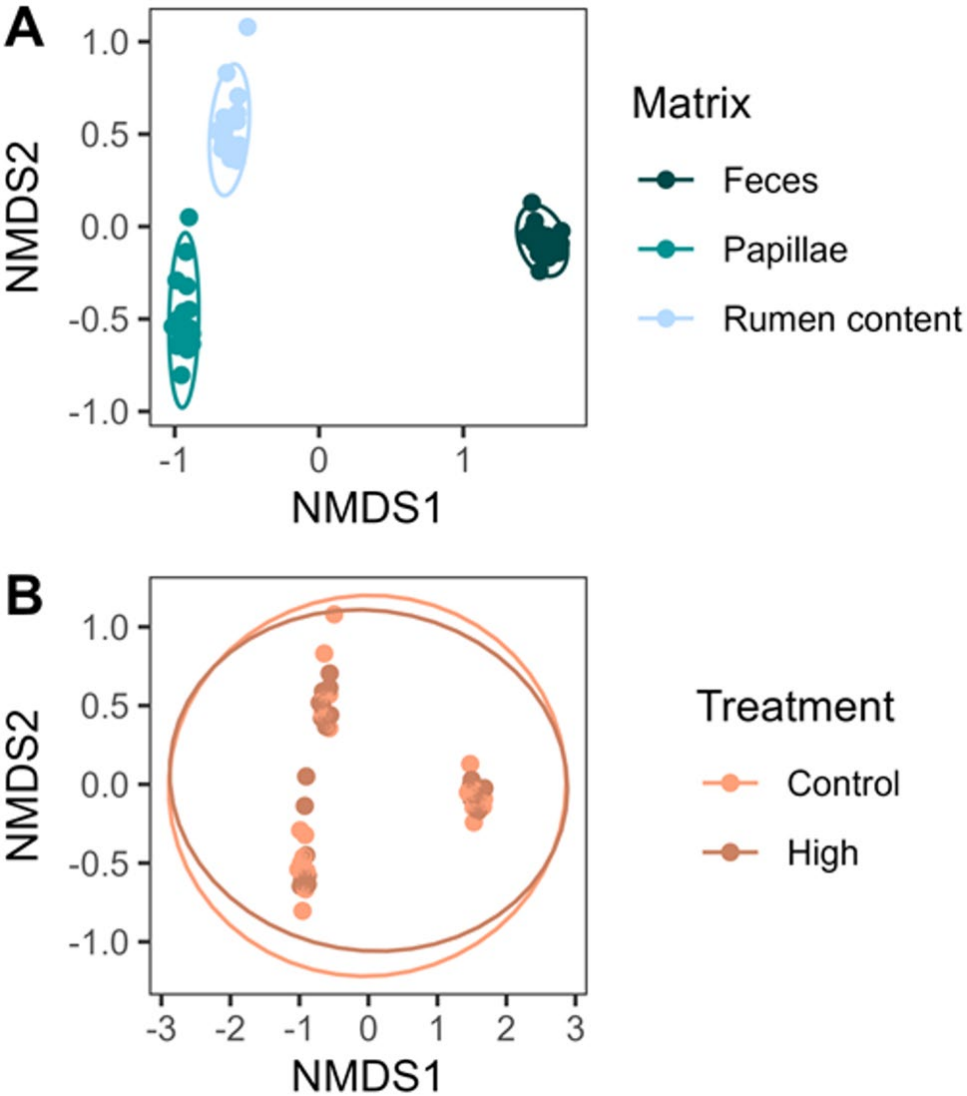
Beta-diversity analysis using bray-curtis dissimilarities between **(A)** matrices or **(B)** treatment. Stress value = 0.073

Feces, papillae and whole rumen content were sequenced using targeted 16 S rRNA amplicon sequencing to assess microbial diversity. After quality control, there were 4,499 operational taxonomic units (OTUs) found with a mean frequency of 19,869 sequence reads per sample. No effect of treatment was seen on the richness (observed features), diversity (Faith PD), nor evenness (Shannon) in any of the sampled matrices (*p* > 0.05). Analysis of beta-diversity using Bray-Curtis dissimilarities revealed clustering according to the matrix (Fig. 1A; *p* = 0.001, F = 32.42, R^2^ = 0.45), but not based on feeding treatment (Fig. 1B; *p* = 0.961, F = 0.35, R^2^ = 0.0075).

### Taxonomic changes

**Fig. 2:**
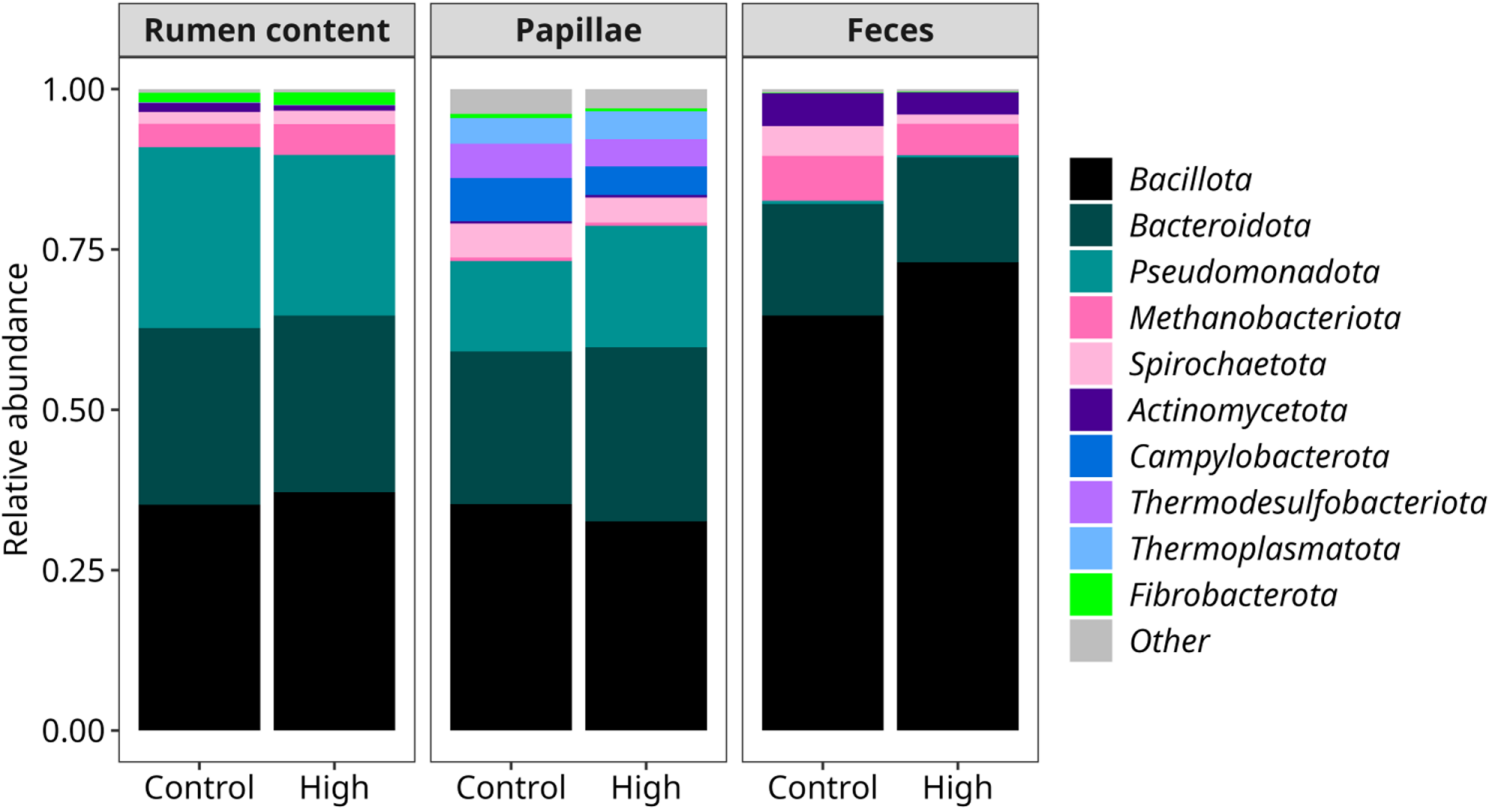
Prokaryotic taxonomic changes at the phylum level. Split by matrix (rumen content, papillae or feces) and by dietary treatment (Control or High)

Prokaryotic composition was analyzed from whole rumen content, papillae-adherent populations and fecal samples, where relative abundances at the phylum level are presented in Fig. 2. The type of matrix had an impact (*p* < 0.05) on all the phyla proportions, except for the *Synergistota* (*p* = 0.09) which accounted for the lowest relative abundance with less than 0.01%. Feces showed different microbial patterns with higher abundances of *Bacillota* (69.0% vs. 35.1% in papillae and 40.3% in rumen) and *Actinomycetota* (5.9% vs. 0.5% in papillae and 1.5% in rumen), as well as lower abundances of *Pseudomonadota* compared to the other two matrices (0.4% vs. 25.2% in papillae and 26.8% in rumen). *Halobacteriota* showed an interaction between matrix and treatment, being significantly more abundant in papillae (Control: 6.7%, High: 3.8%, *p* interaction = 0.0043) and nearly non-detectable in other matrices. *Elusimicrobiota* tended to be less abundant (*p* = 0.07) in the rumen and feces of cows fed High mineral (0.75% and 3.3%) compared to Control (1.19% and 5.07%, respectively). Analysis showed that a number of bacterial genera showed a interaction between matrix and treatment, so analysis was performed for each matrix independently. Some bacterial genera from each sampled matrices were also impacted by the dietary treatment as shown in Table 4. In the papillae, *Desulfovibrio* (*p* = 0.025) *Defluvitaleceae UCG001 (p* = 0.052), and *Treponema* (*p* = 0.010) were more abundant in the Control treatment and *Campylobacter* showed a tendency towards being increased in the Control treatment as well (*p* = 0.059). Communities of the rumen content exposed to High TM showed an increased abundance of *Ruminococcus* (*p* = 0.013), whereas *Methanosphaera* (*p* = 0.015) was increased in the Control group. Cows fed the High diet had lower proportions of *Methanosphaera and Treponema* in their feces (*p* = 0.013), matching that seen in the other matrices.

**Table 4:** Differential relative abundance of bacterial groups from the rumen papillae and content, and fecal matrices based on surplus trace mineral feeding in dairy cattle

Matrix	Treatment			
	Control	High	SEM	*P*-value
**Papillae**				
*Campylobacter*	6.72	3.83	1.443	0.059
*Defluvitaleceae UCG011*	0.231	0.166	0.0325	0.052
*Desulfovibrio*	1.92	1.06	0.431	0.025
*Treponema*	4.86	2.97	0.944	0.010
**Rumen content**				
*Methanosphaera*	0.439	0.291	0.0741	0.015
*Ruminococcus*	2.20	3.10	0.453	0.013
**Feces**				
*Methanosphaera*	0.609	0.374	0.1173	0.013
*Treponema*	4.63	1.86	1.388	0.013

^*^Cows in the Control treatment were fed recommended levels of minerals, whereas cows in the High treatment were fed surplus levels of cobalt, manganese and zinc

### Correlation between mineral and microbes

In the rumen content, the level of phosphorus was highly correlated with both alpha diversity (Shannon and Simpsons; Table 5) as well as the phyla *Bacteroidota*, *Bacillota*, *Patescibacteria*, *Actinomycetota*, *Pseudomonadota* (negative) and *Spirochaetota* (Table 6). Potassium feeding had a negative correlation to Elusimicrobiota and calcium exhibited a marked positive impact on the presence of the *Verrucomicrobiota* phylum. This translated to many highly correlated genera including *Acetitomaculum*, *Prevotella* (negative), *Ruminococcus*, *Shuttleworthia* (negative), *Treponema*, and *Xylanibacter* (Table 7). Copper was moderately positively correlated with *Halobacteriota* but not with any of the top 50 most abundant genera [see Additional file S3]. Similarly, Zn was not correlated with any microbes in the rumen content.

**Table 5:** Impact of measured mineral concentrations on microbiome alpha diversity indices in the gut of lactating dairy cows

	Rumen content				Papillae		Feces			
	Shannon		Simpson		Simpson		Observed OTU		Chao1	
	*r*	*p*-value	*r*	*p*-value	*r*	*p*-value	*r*	*p*-value	*r*	*p*-value
P	0.81	< 0.001*	0.78	< 0.001*	0.07	0.795	0.17	0.524	-0.021	0.940
K	0.34	0.200	0.26	0.339	-0.47	0.064	-0.49	0.054*	-0.53	0.035*
Ca	0.44	0.090	0.39	0.137	-0.46	0.070	0.35	0.178	0.32	0.231
Mg	0.31	0.240	0.24	0.368	-0.51	0.044*	0.31	0.242	0.30	0.259
Fe	0.25	0.345	0.21	0.431	-0.30	0.264	0.08	0.778	0.09	0.729
Co	0.28	0.289	0.20	0.451	-0.43	0.094	0.32	0.230	0.30	0.264
Cu	-0.31	0.249	-0.34	0.196	-0.39	0.131	0.13	0.644	0.19	0.471
Mn	0.41	0.119	0.36	0.172	-0.25	0.356	0.35	0.186	0.29	0.279
Zn	-0.01	0.983	-0.02	0.931	-0.09	0.729	0.26	0.324	0.27	0.316

In the papillae, the level of K and Mg were highly negatively correlated with the presence of *Verrucomicrobiota* (Table 6). The level of iron was highly correlated with the presence of *Prevotellaceae* NK3B31 group, *Prevotella* UCG 004, UCG 005 and *Xylanibacter* (Table 7). Copper was positively correlated to *Leyella*, *Prevotella* UCG 004, and *Succinivibrionaceae* UCG 001 (Table 7). Zn was moderately correlated to the presence of *Corynebacterium* and *Prevotellaceae* Ga6A1 group [see Additional file S4].

**Table 6:** Significant correlations between measured minerals and prokaryotic phylum across rumen content, rumen papillae and fecal matrices

Matrix	Mineral		Phylum	*r*	*p*-value
Rumen content	P	*Fibrobacterota*		0.66	0.005
		*Bacillota*		0.70	0.002
		*Spirochaetota*		0.68	0.004
		*Patescibacteriota*		0.68	0.005
		*Bacteroidota*		0.71	0.002
		*Actinomycetota* *Verrucomicrobiota* *Methanobacteriota*		0.68 0.52 0.64	0.004 0.04 0.007
		*Pseudomonadota*		-0.79	0.003
	K	*Elusimicrobiota* *Thermodesulfobacteriota*		-0.67 0.61	0.026 0.01
	Ca	*Verrucomicrobiota*		0.79	< 0.001
	Cu	*Chloroflexota*		0.50	0.047
		*Halobacteriota*		0.53	0.033
	Mn	*Actinomycetota*		0.69	0.038
Rumen papillae	P	*Elusimicrobiota*		-0.53	0.035
	K	*Verrucomicrobiota*		-0.68	0.003
	Mg	*Verrucomicrobiota*		-0.66	0.005
		*Planctomycetota*		-0.56	0.023
Feces	P	*Bacteroidota*		-0.54	0.039
	K	*Bacterioidota*		0.52	0.046
		*Actinomycetota*		0.55	0.035
	Ca	*Bacillota*		0.69	0.004
	Fe	*Campylobacterota*		0.55	0.035
	Co	*Bacillota*		0.61	0.017
	Mg	*Actinomycetota*		-0.53	0.041
	Zn	*Bacillota*		0.57	0.027
	Mn	*Bacillota*		0.58	0.023

In the feces, P and Mg were highly negatively correlated to the presence of *Verrucomicrobiota* (Table 6). Similar to the rumen, Iron feeding was highly correlated to *Prevotellaceae* NK3B31 group, *Prevotellaceae* UCG 004, UCG 005 and *Xylanibacter* (Table 7). Copper was moderately correlated to *Prevotellaceae* UCG 004, Shuttleworthia, and Succinivibrionaceae UCG 001 [see Additional file S5]. Zn was moderately correlated to *Corynebactrium*, and *Prevotellaceae* Ga6A1 group, as well as negatively correlated to *Syntrophococcus* [see Additional file S5].

**Table 7:** Correlations of interest between measured minerals and bacterial genera across rumen content, rumen papillae and feces

Matrix	Mineral	Group	*r*	*p*-value
Rumen content	P	*Acetitomaculum*	0.71	0.002
		*Christensenellaceae* R 7	0.68	0.004
		*Dialister*	-0.72	0.002
		*Fibrobacter*	0.68	0.004
		*Lachnospiraceae* NK3A20 group	0.76	< 0.001
		*Methanobrevibacter*	0.64	0.007
		*Methanosphaera*	0.64	0.007
		*NK4A214group*	0.75	< 0.001
		*Oribacterium*	-0.65	0.006
		*Prevotella*	-0.79	< 0.001
		*Prevotellaceae* Ga6A1 group	0.71	0.002
		*Prevotellaceae* NK3B31 group	0.63	0.009
		*Prevotellaceae* UCG 001	0.66	0.006
		*Rikenellaceae* RC9 gut group	0.78	< 0.001
		*Ruminococcus*	0.75	< 0.001
		*Shuttleworthia*	-0.78	< 0.001
		*Succinivibrionaceae* UCG 001	-0.79	< 0.001
		*Treponema*	0.74	0.001
		*Xylanibacter*	0.78	< 0.001
	K	*Succiniclasticum*	0.70	0.003
		*Syntrophococcus*	-0.65	0.006
	Ca	*Defluviitaleaceae* UCG 011	-0.65	0.006
	Fe	*Defluviitaleaceae* UCG 011	-0.70	0.002
	Mn	*Methanobrevibacter*	0.69	0.035
Papillae	P	*Syntrophococcus*	-0.61	0.012
	K	*Prevotellaceae* UCG 001	-0.61	0.013
	Mg	*Prevotellaceae* UCG 001	-0.65	0.006
	Fe	*Prevotellaceae* NK3B31 group	0.88	< 0.001
		*Prevotellaceae* UCG 004	0.67	0.005
		UCG 005	0.67	0.005
		*Xylanibacter*	0.77	< 0.001
	Cu	*Leyella*	0.65	0.006
Feces	P	*Bacteroides*	-0.66	0.007
	K	*Howardella*	-0.63	0.011
		*Olsenella*	-0.65	0.009
		*Syntrophococcus*	-0.72	0.003
	Ca	*Olsenella*	-0.65	0.009
		*Shuttleworthia*	0.61	0.017
		*Syntrophococcus*	0.65	0.009
	Fe	*Howardella*	0.65	0.009
	Mn	*Lachnospiraceae* NK3A20 group	0.61	0.015

Analysis of metabolism pathways showed little differences between the two dietary treatments, where small variances were seen for the short-chain fatty acids pathway [see Additional file S6]. Metabolism pathways in metagenome-assemble genomes from cows’ feces fed recommended (control) or surplus levels (high) of trace minerals. The abundance increases with the red intensity.

Figure representing metabolism pathways in metagenome-assemble genomes from cows’ feces.

### Resistome

**Fig. 3:**
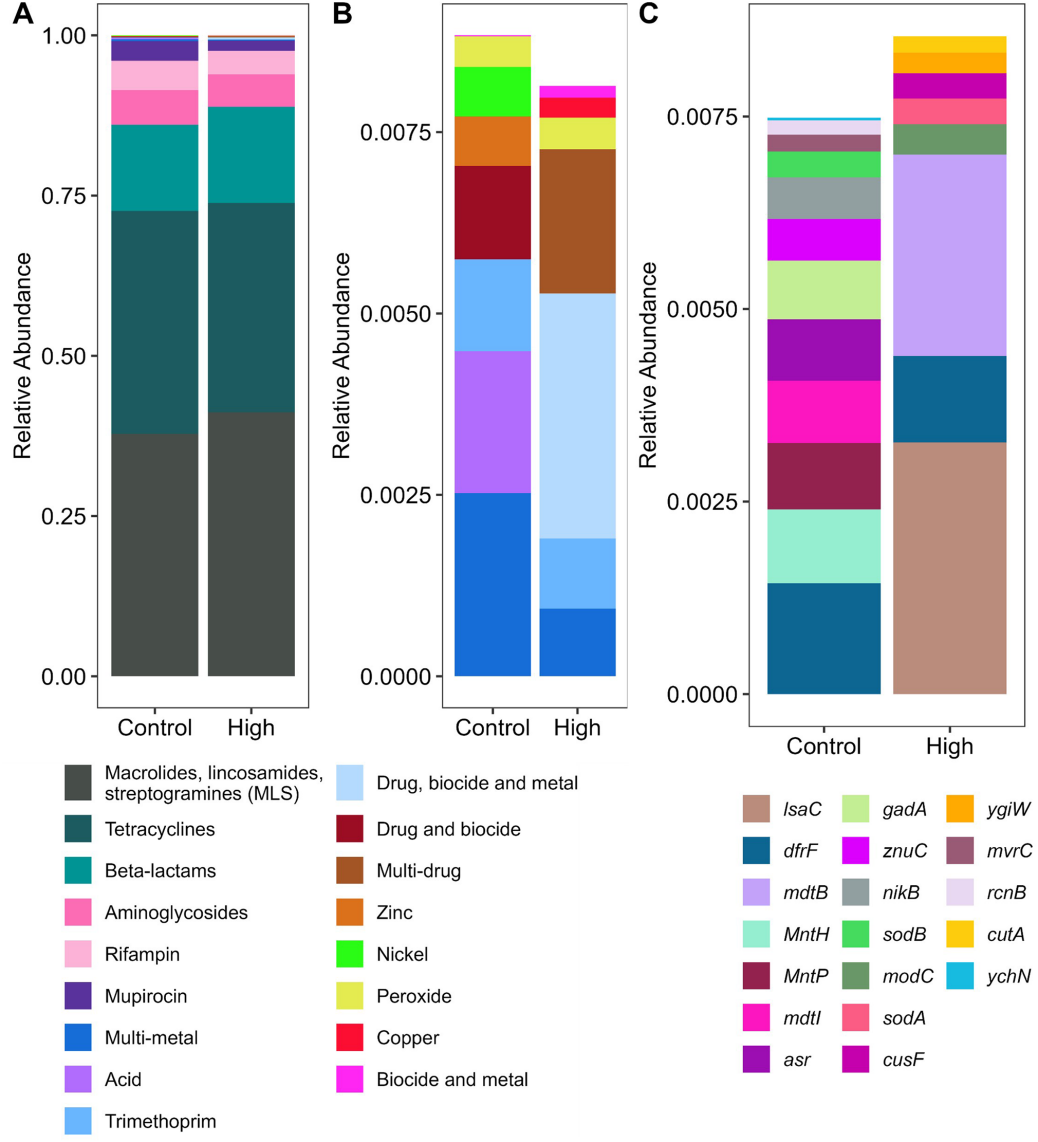
Feces resistome of cows fed recommended (Control) or surplus levels (High) of trace minerals. Abundances were transformed using cumulative sum scaling (CSS). **(A)** Representation of all the antimicrobial classes. **(B)** Focus on the low antimicrobial classes accounting for less than 1% of the total population represented in panel A (top 6 removed). **(C)** Antimicrobial resistance genes represented in the low antimicrobial classes

The fecal resistome was assessed by shotgun metagenomics and was highly similar for both groups as shown in Fig. 3A, where resistance to macrolides, lincosamides, streptogramines (MLS), tetracyclines and beta-lactams were predominant. Although the MaAsLin2 analysis did not reveal any significant differences between the two groups, some dissimilarities could be observed for the low relative abundance genes (Fig. 3B), where acid (*gadA*, *ASR*), drug and biocide (*mvrC*, *mdtI*), zinc (*znuC*) and nickel (*nikB*) resistance were only detected in the Control group. Drug, biocide and metal (*mdtB*) as well as multi-drug (*lsaC*) resistance genes were only present in the feces of cows fed the High diet (Fig. 3C).

**Fig. 4:**
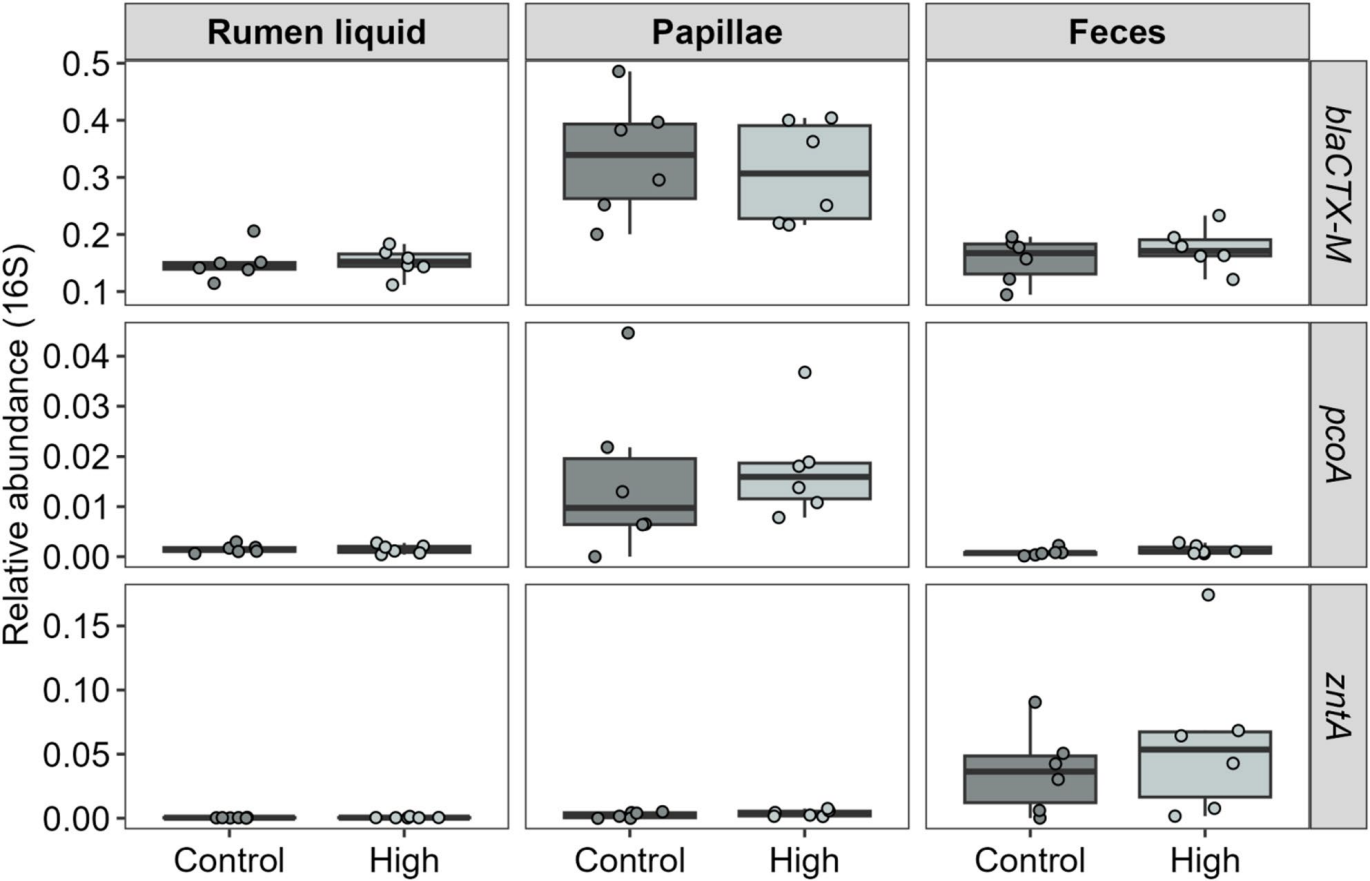
Quantification of antimicrobial resistance genes in rumen liquid, papillae and feces when lactating dairy cows where fed recommended (Control) or surplus levels of dietary minerals (High). Abundance of genes is relative to the *16Sr RNA* quantification

Using ddPCR, *bla*_CTX−M_, *pcoA* and *zntA* were quantified in rumen liquid, papillae and feces samples (Fig. 4). The *bla*_CTX−M_ genes are among the most prevalent extended spectrum β-lactamase encoding genes in the gut of livestock animals, in addition to being clinically important. The product of the *pco* operon performs direct detoxification and strongly correlates with functional copper tolerance (10.1099/00221287-131-4-939). The *pco* cluster is plasmid-borne and is found in *Enterobacteriaceae* (PMID: 26893455). The *zntA* gene was selected as it is tightly linked to zinc resistance. It encodes a transporter mediating the active export of cytoplasmic Zn2+ (10.1073/pnas.94.26.14326). Even though no significant differences were observed between the two groups, notable differences were seen between the matrices. While papillae samples showed higher abundances of both *bla*_CTX−M_ (*p* < 0.0001) and *pcoA* (*p* = 0.0018), feces showed higher abundance of *zntA* compared to the other matrices (*p* < 0.0001).

## Discussion

Although trace minerals (TM) are essential to maintain the metabolic health and milk productivity of dairy cows, feeding above minimal requirements is common practice [46], requiring a better understanding of the interactions between diet, microbial health, animal production, and AMR. This is specifically an area of concern as some TMs, such as copper and zinc, are known to exhibit antimicrobial effects [3] that can contribute to the dissemination of antimicrobial resistance (AMR) including in livestock animals [5, 6]. In commercial dairy nutrition, however, trace minerals are supplemented as part of a complete mineral premix rather than individually. Consequently, modifying the inclusion rates of specific TMs (e.g., Co, Mn, Zn) occurs within the context of the broader dietary mineral profile. The primary focus of this study was on the effects of surplus cobalt, manganese, and zinc on microbial populations across distinct gastrointestinal matrices in lactating cows fed TM above recommended concentrations, particularly in relation to antimicrobial resistance genes (ARGs) and metal resistance genes (MRGs). In this study cannulated lactating dairy cows were fed either a basal (Control) or a surplus of Co, Mn and Zn (High) in the diet, then papillae, rumen content and feces samples were analyzed to assess changes in the prokaryotic community and its associated resistome, using sequencing technologies (targeted and shotgun metagenomics) as well as ddPCR. The impact of those diets on the cow performance and the excretion of TMs were previously published, where it was shown that diet did not impact the lactational performance, but did lead to increased fecal excretions of cobalt, manganese and zinc [17].

One of the limitations of studying tissue-adherent populations in the gastrointestinal tract is that biopsies are more invasive and therefore sampling is not as extensive as other matrices. As samples for tissue-adherent microbes in this study were collected at different time points, this could contribute to variation in microbial composition. However, as it is well established that various gut matrices are phylogenetically distinct, as such, the goal of this project was to understand how dietary treatments differientially impact these distinct populations, rather than to evaluate temporal dynamics across matrices. Therefore, samples from each matrix were statistically analyzed separately to remove day of sampling as a factor. As expected, the prokaryotic composition was different depending on the gut location. *Bacillota*, *Bacteroidota* and *Pseudomonadota* are normal habitants of the ruminant gastrointestinal system [47–49]. Different abundances observed at the phylum level can generally be attributable to pH variations and substrate availability based on diet, which differ throughout the gastrointestinal tract [50], leading to competition and cross-feeding between the various prokaryotic community members. For example, when the rumen is exposed to non-digestible carbohydrates, it can lead to an increase of *Pseudomonadota* in comparison with fecal samples [49]. Thomas et al. studied the impact of antibiotic supplementation on the gut microbiome and resistome of cattle, and similar to the current study, observed significant differences in the prokaryotic composition between the rumen, colon and cecum despite minimal influences on the prokaryotic taxonomy and ARGs found [51]. The results of the current study showed that the influence of TM on the prokaryotic communities was also different in each gut location, which was expected, since most mineral absorption occurs in the small intestine [52], and therefore the largest amounts of minerals would be available to prokaryotic members of the rumen. This could explain why the majority of correlations between minerals and prokaryotic phylum took place in the rumen content.

The analysis of rumen ecology with a broader lense is critical in understanding how nutrition impacts the various members of the community and their potential interactions. Therefore, this study was performed to look at the prokaryotic community (bacteria and archaea) using the V4 region of 16 S RNA gene. The aim of this analysis was provide baseline data to understand how minerals could potentially impact methanogens, in addition to the more well studied bacterial populations. While microbial community compositions were highly similar between both treatments for each of the matrices, small variations in relative abundances were observed. In the present study, *Spirochaetota* were more abundant in the papillae and feces of cows fed the control diet, whereas *Bacillota* were increased in the feces of cows fed higher concentrations of TM. Previously, increases in *Bacillota* and *Actinomycetota* were also noted in colon of cattle fed with nano zinc oxide [53, 54], variations in results to the present experiment could reflect bioavailability as the present trial used sulfate source of TM. At the genera level, cows fed high concentrations of TM had increased levels of *Ruminococcus* in their rumen content. A hypothesis for this is that diets with different TM compositions may have triggered different metabolic pathways in the gut, linked to nutrient availability or bacterial cross-feeding. This hypothesis is based on data from Marchand et al. [17], where there were observed modifications in proteolysis according to TM feeding, and increased proportions of rumen isovalerate in cows fed surplus TM. To assess this hypothesis, metabolism pathways were analyzed in the metagenomes. Although small changes were observed between the two groups, particularly for the short-chain fatty acids, the analysis was inconclusive due to poor sample size (*n* = 2/group). As many enzymes are known to have metals as cofactors, Zn, Fe and Mn being the most prevalent [55], this could explain the correlations between mineral and microbes. *Ruminococcus* is implicated in rumen fiber degradation, even highlighted as one of the most important bacteria capable of degrading cellulose [56]. In a study conducted in lambs, it was shown that high levels of Mn (182.7 and 184 ppm) could impact levels of eubacteria and enzyme activities in the rumen, specifically, increase the activity of carboxymethyl-cellulase [57]. *Ruminococcaceae* are known to adapt rapidly to diet modifications and to play a role in carbohydrate metabolism [58]. Correlations between TM and *Ruminococcus* could also be explained by enzymatic cofactors. These results conclude that due to the ubiquitous usage of TM, the gut microbiota shows great stability and adaptation to its feeding level.

Using shotgun metagenomics, the fecal resistome of lactating dairy cows was characterized considering its implications for environmental and human health. The results showed that most resistance was conferred towards MLS and tetracyclines, which is in line with previous literature [59]. While no differences were seen based on short-term surplus TM feeding, this information adds to a baseline of understanding for ARG presence in the gut of lactating cattle. Improved comprehension of ARGs in livestock is critical for understanding the greater impacts of management practices (feed, stress and handling, manure containment and spreading), on the One Health continuum. Specific ARGs were also quantified using ddPCR. Once again, there was no difference between the two groups, but abundances of genes was modulated by the matrix type. Interestingly, the level of those ARGs did not match with the correlations between TM and bacteria. z*ntA* confers resistance to Zn, however, no correlations between Zn and microbiome alpha diversity or phylum in the feces were significant. A similar situation was observed for the copper resistant gene *pcoA*, although a few bacterial groups were positively correlated with Cu in the rumen papillae. This could indicate that these genes are likely carried by a diverse group of bacteria, rather than being limited to a specific species, and that phenotypic resistance requires timely expression of multiple genes. However, it is critical to note that the limited number of metagenomic samples and the pooling strategy may have reduced the sensitivity required to capture the full complexity of the resistome. While samples were pooled within treatment, it is well understood that individual animal variation in the ruminant microbiome is often sufficiently large to mask treatment effects [60]. Consequently, differences in the ARG and MRG abundance may not have been resolved in the present study. The primary objective of the metagenomics analysis was to identify higher-level changes in the resistome profile associated with excessive mineral supplementation, using complementary techniques to support broader microbial analysis. Future studies should focus on individual replicates to improve resolution, as well as targeted resistome enrichment strategies to provide detailed information regarding the impact of trace minerals supplementation on metal resistance dynamics.

## Conclusions

This study aimed to understand the implications of short-term feeding of surplus levels of TMs to lactating dairy cows on their gut microbiome and resistome, in order to limit their impact on the gastrointestinal tract and ultimately the environment. Using targeted amplicon sequencing, shotgun metagenomics, and ddPCR, it was shown that surplus sulfate TM (Co, Mn, Zn) had a minimal impact on the microbiome and resistome of rumen, papillae and fecal samples from lactating dairy cattle after 31 days. Although changes were noted for specific microbes in specific matrices, no significant changes to the presence of key ARGs were detected. This work highlights the plasticity of the gut microbiome of the lactating dairy cows and justifies further research to understand long-term effects of surplus TM feeding on animal health, microbial evolution and bioaccumulation.

## Supplementary Information

Below is the link to the electronic supplementary material.

Supplementary Material 1: Sequencing depth and filtering of 16S samples. Table showing the number of sequences (raw, trimmed and filtered) for each 16S sample presented in the study.

Supplementary Material 2: Number of reads per metagenome after each quality filtering step. Table showing the number of reads (raw, trimmed and decontaminated) for each metagenome presented in the study.

Supplementary Material 3: Correlations between mineral and microbes in rumen content. Table showing correlation analysis between mineral content of diet and microbes associated with the rumen.

Supplementary Material 4: Correlations between mineral and microbes in rumen papillae. Table showing correlation analysis between mineral content of diet and microbes associated with the papillae.

Supplementary Material 5: Correlations between mineral and microbes in feces. Table showing correlations between mineral and microbes in the feces.

Supplementary Material 6: Metabolism pathways in metagenome-assemble genomes of feceal microbiota. Graphic of the metabolic pathways from metagenome-assemble genomes from cows’ feces fed recommended (control) or surplus levels (high) of trace minerals. The abundance increases with the red intensity.

## Data Availability

Sequences have been deposited to the NCBI Sequence Read Archive, where the BioProject can be found under accession number PRJNA1314803.
