# Supplementary material for: Impact of differential dietary concentrations of cobalt, manganese and zinc on gastrointestinal microbiome and resistome of lactating dairy cattle
Source: Anim Microbiome. 2026 Mar 25;8:61. doi: 10.1186/s42523-026-00554-9 (PMC13137713; doi:10.1186/s42523-026-00554-9)
Supplement: Supplementary file 2 — Supplementary Material 2: Number of reads per metagenome after each quality filtering step. Table showing the number of reads (raw, trimmed and decontaminated) for each metagenome presented in the study. [file 42523_2026_554_MOESM2_ESM.docx]

**Table S1**. Number of reads per metagenome after each quality filtering step.

|  |  | Number of reads | | |
| --- | --- | --- | --- | --- |
| Period | Treatment | Raw | Trimmed | Decontaminated |
| 1 | Control | 77 623 877 | 75 834 328 | 75 720 330 |
| 1 | High | 80 150 480 | 79 074 717 | 78 885 774 |
| 2 | Control | 75 387 064 | 73 935 339 | 73 871 099 |
| 2 | High | 75 520 444 | 73 915 940 | 73 822 501 |
