# Supplementary material for: Impact of differential dietary concentrations of cobalt, manganese and zinc on gastrointestinal microbiome and resistome of lactating dairy cattle
Source: Anim Microbiome. 2026 Mar 25;8:61. doi: 10.1186/s42523-026-00554-9 (PMC13137713; doi:10.1186/s42523-026-00554-9)
Supplement: Supplementary file 6 — Supplementary Material 6: Metabolism pathways in metagenome-assemble genomes of feceal microbiota. Graphic of the metabolic pathways from metagenome-assemble genomes from cows’ feces fed recommended (control) or surplus levels (high) of trace minerals. The abundance increases with the red intensity. [file 42523_2026_554_MOESM6_ESM.pptx]

## Slide 1
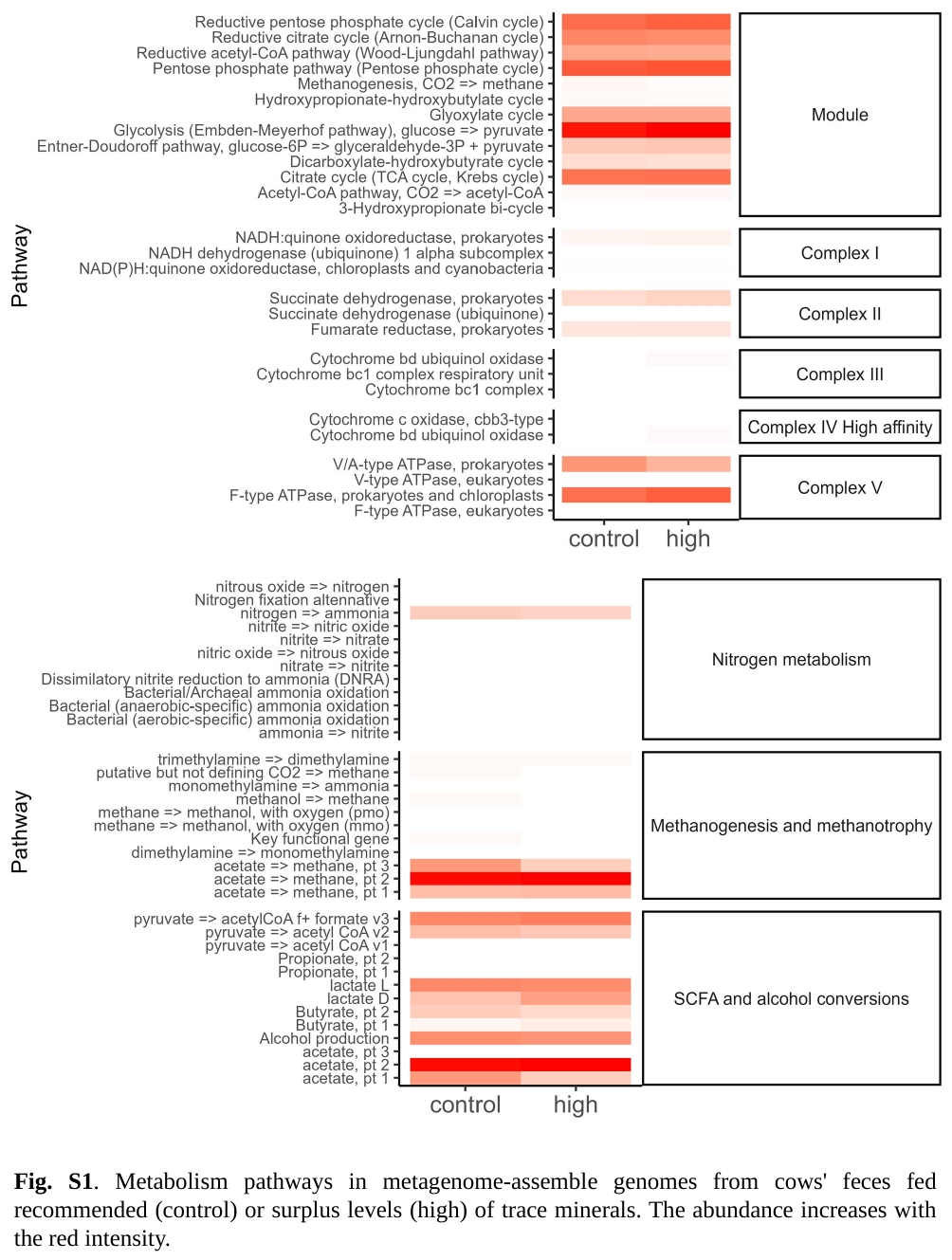

Fig. S1. Metabolism pathways in metagenome-assemble genomes from cows' feces fed recommended (control) or surplus levels (high) of trace minerals. The abundance increases with the red intensity.
